# Web-Based Health Information Seeking Among African American and Hispanic Men Living With Chronic Conditions: Cross-sectional Survey Study

**DOI:** 10.2196/26180

**Published:** 2021-07-14

**Authors:** Ledric D Sherman, Kirby Goidel, Caroline D Bergeron, Matthew Lee Smith

**Affiliations:** 1 Department of Health & Kinesiology Texas A&M University College Station, TX United States; 2 Public Policy Research Institute & Department of Political Science Texas A&M University College Station, TX United States; 3 Public Health Agency of Canada Ottawa, ON Canada; 4 Department of Environmental and Occupational Health Center for Population Health and Aging Texas A&M University College Station, TX United States

**Keywords:** minority men, online information seeking, chronic disease, communication with health care providers, mobile phone

## Abstract

**Background:**

Previous research has identified disparities in seeking and using web-based health information to inform health-related behaviors. Relatively few studies however have examined the correlations between web-based health information seeking and use based on race, gender, age, and the presence of chronic health conditions.

**Objective:**

In this study, we identify factors associated with seeking and using web-based health information among a uniquely vulnerable and intersectional population—middle-aged and older (40 years and older) African American and Hispanic men living with one or more chronic conditions.

**Methods:**

Survey responses were collected from a purposive sample of African American and Hispanic men using Qualtrics web-based survey management software. To qualify for inclusion in the study, respondents had to identify as African American or Hispanic men, report having at least one chronic condition, and be aged 40 years and older. A series of binary logistic regression models was created using backward elimination. Statistical significance was determined at *P*<.05 for all analyses.

**Results:**

Web-based health information seeking among African American and Hispanic men is a function of education, the presence of multiple chronic conditions, frustration with health care providers, internet use, and the perceived reliability of web-based health information. The use of web-based health information to inform interactions with health care providers was more common among African American and Hispanic men, who rated their health as relatively good, perceived barriers to care, used technology regularly, and took more daily medications.

**Conclusions:**

Understanding the factors that influence African American and Hispanic men seeking web-based health information may help improve the care and treatment of chronic conditions. African American and Hispanic men seek web-based health information as a substitute for routine care and to inform their discussions with health care providers.

## Introduction

### Background

Information seeking encompasses the act of accumulating information to gain clarity or affirm knowledge about a specific topic [1]. Well-informed patients maintain a sense of control over their illness and are better able to cope with uncertainties related to outcomes and treatments [2-5]. Correspondingly, knowledgeable patients engage with medical providers in planning their care, managing their treatments, and adapting more readily to therapeutic schedules [6,7]. Insufficient health information, in contrast, can have unfavorable health consequences [2,8].

Health-related information can be obtained from supportive social networks, health care providers, and the media, including the internet, television, radio, books, or magazines [9,10]. Web-based health resources provide an *optimal way to disseminate health information* because there is the “immediacy of information access, the accessibility at any time of the day or night, the potential continual updating of information and the wider range of information available” [11]. However, disparities exist in terms of who pursues or seeks web-based health information and how the information is used to inform subsequent interactions with health care providers [7,12,13]. Although men use the internet more often than women, they use it less frequently to seek health information [14]. Men are also less likely to seek routine medical care than women and therefore have fewer opportunities to discuss web-based health information with their health care providers [15]. Race and ethnicity are also associated with internet-based health information seeking [16,17]. Historically, health information seeking was less common among racial and ethnic minorities because of limited internet access and lower health literacy skills [12,13,18-20]. However, recent research has suggested that these differences may be dwindling, with African Americans relying more heavily on web-based health information for health care [21,22].

Other factors associated with web-based information include education [19], self-reported health status [23,24], time spent with medical providers and frustrations in communicating with these providers [25,26], internet use [23,27], and the perceived reliability of web-based health information [28]. Previous research does not provide enough clarity on how these factors might affect internet-based information-seeking behaviors of African American and Hispanic men with chronic conditions; however, there is reason to expect some differences. Some studies have examined web-based health information seeking by race [29,30] or by sex, specifically for men with chronic diseases [31], but did not focus specifically on African American and Hispanic men with chronic conditions. This population has been found to experience important barriers to disease self-management [32], have less access to health insurance and preventative care [33], have higher rates of preventable hospitalizations [34], and are more likely to die from their chronic conditions compared with non-Hispanic White men [35]. Seeking and using credible web-based health information may represent an important health-promoting activity.

In this paper, we seek to understand web-based health information seeking among African American and Hispanic men. Our motivation is both substantive and methodological. First, African American and Hispanic men are less likely to seek preventative care and treatment, which subsequently affects health outcomes. Understanding the factors that lead to seeking web-based health information may lead to better health outcomes. Second, African American and Hispanic men are hard-to-reach populations in survey research, meaning that they are often underrepresented in probability-based samples.

### Objective

In this 2-phase study, we contribute to the existing literature by investigating web-based health information seeking and use among African American and Hispanic men aged 40 years and older with one or more chronic conditions. In phase 1, we identify factors associated with seeking web-based health information in the past year about (1) a specific disease or medical problem and (2) medical treatments and procedures. Then, in phase 2, we identify factors associated with discussing web-based health information with primary medical professionals only among those men who sought health information on the internet and had a routine physician visit within the past year.

## Methods

### Overview

Due to increased costs and declining response rates, scholars increasingly rely on web-based panels when studying hard-to-reach or intersectional populations [36]. African American and Hispanic men with chronic conditions and aged 40 years or older, for example, are relatively small segments of the overall population, making random selection via probability sampling costly and inefficient. Due to distrust of medical providers, African American and Hispanic men are often less responsive to requests to participate in health-related research [37].

With this in mind, the sample in this study was designed using Qualtrics (Systems, Applications, and Products in Data Processing Societas Europaea) web-based panels to identify African American and Hispanic men aged 40 years and older with at least one chronic health condition. We used the Checklist for Reporting Results of Internet E-Surveys for web-based surveys in our description of data collection [38]. Qualtrics web-based panels are opt-in research panels ideal for studies targeting hard-to-reach populations. Qualtrics panels provide access to previously identified research participants with known characteristics, and panel participants are recruited and compensated for their participation by Qualtrics. Potential participants were directed to the programmed survey where they were provided with a description of the study and information relating to informed consent. The tradeoff for cost effectiveness using Qualtrics is that the sample might not be representative of the target population.

The survey questionnaire was constructed by the authors who identified validated questions from previous research related to web-based health information seeking and other health-related behaviors. An initial draft of the survey was carefully reviewed by experts in the field who were not a part of the research team and made suggestions for inclusion (or exclusion) of specific items. The final data were carefully reviewed by the research team, eliminating questionable responses (eg, respondents who completed the survey too quickly). In addition, filter questions for age, race, and the presence of one or more chronic conditions were used to further qualify potential respondents and ensure that only qualified respondents completed the survey questionnaire. The survey instrument included a wide range of health-related attitudes and behaviors. Overall, data were collected from 2028 men who met the inclusion criteria. This study was approved by the Institutional Review Board (#2018-1684) of Texas A&M University.

Inspired by Pettus et al [39], who examined internet use and web-based health information seeking among older women in a 2-phase study, we modeled this study to focus on web-based health information seeking among middle-aged and older men. To be included in phase 1 of our analytic sample, men also had to report using the internet within the past 2 weeks. Men who did not meet this criterion were excluded from phase 1 analyses. The 2 dependent variables in phase 1 assessed web-based health information seeking and use, which was measured using 2 items. First, participants were asked if they had looked for information on the internet about “a specific disease or medical problem.” Response choices for this item were “yes” and “no.” Second, participants were asked if they had looked for information on the internet about “a certain medical treatment or procedure.” Response choices for this item were “yes” and “no.”

**Figure 1:**
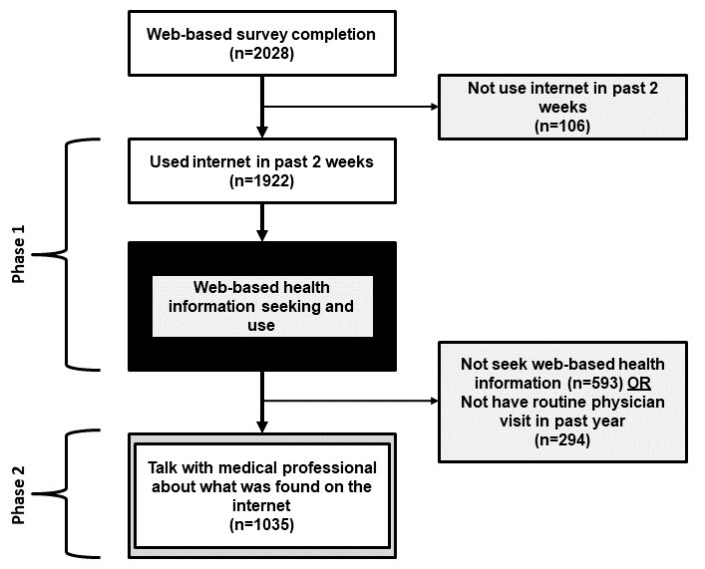
Study flow by analysis phases.

Building upon findings in phase 1, to be included in phase 2 of our analytic sample, men must have reported “yes” to looking for information on the internet about “a specific disease or medical problem” or “a certain medical treatment or procedure. In addition, to avoid confounding the results with issues of health care access, these minority men must have reported having a routine physician visit in the past year to be included in phase 2. Men who did not meet this criterion were excluded from phase 2 analyses. The dependent variable in phase 2 assessed whether men shared findings from their web-based health information seeking with medical professionals. More specifically, participants were asked if they spoke with a medical professional about what they found on the web. Response choices for this item were “yes” and “no.” Figure 1 illustrates the participant flow across both study phases based on the inclusion criteria.

We modeled web-based health information seeking as a function of demographics (age, race, education, marital status, and number of household members), health-related behaviors and status (number of chronic conditions, number of daily medications, having a routine physician visit in the past year, and self-reported health status), available resources for managing care (receiving help to manage care, ability to self-manage diseases, perceived barriers to care, health care frustrations, and participation in programs to prevent or manage chronic illness), and technology use and credibility (use of technology and reliability of web-based health information). We provide brief descriptions of each of these below.

### Demographics

Age was measured in years, with all respondents reporting that they were aged 40 years or older. Race is a dichotomous variable indicating whether the respondent is African American (coded as 0 and serving as the baseline category) or Hispanic (coded as 1). Marital status was measured as a set of dummy variables indicating whether the respondent was single or never married, married or partnered, divorced or separated, or widowed. The number of household members was the total number of people (including the respondent) currently living in the household. The demographic variables in our models were primarily included as controls.

### Health-Related Behaviors

This set of variables included health conditions, regular doctor visits, and self-reported health status. Overall, it is expected that individuals with worse health, meaning more chronic conditions and poor self-reported health status, would be more likely to look for health information on the internet. The number of chronic conditions was calculated using a “check all that apply” list of the following 19 chronic health conditions: (1) asthma, emphysema, chronic breathing problem, or lung problem; (2) arthritis or rheumatic disease; (3) cancer or cancer survivor; (4) chronic pain; (5) depression or anxiety; (6) diabetes; (7) heart disease; (8) high cholesterol; (9) hypertension; (10) kidney disease; (11) memory problem; (12) obesity; (13) osteoporosis; (14) obstructive sleep apnea; (15) schizophrenia or other psychotic disorder; (16) stroke; (17) thyroid problem; (18) urinary incontinence; and (19) another chronic condition not listed. In addition, participants were asked to report the number of different medications taken daily (range 0 to >6), whether they had visited a doctor in the past year (coded 1 if the respondent said yes; 0 otherwise), and a 5-point Likert scale measure of their self-reported health status ranging from poor (coded as 1) to excellent (coded as 0).

### Resources for Managing Care

In addition to health-related behaviors and concerns, individuals with more resources available for managing care should be more likely to seek health information on the internet. This begins with perceptions of whether or not they are receiving the support they need to improve their health and manage their care, measured using a 5-point scale ranging from never (1) to always (5) [23,24]. Due to the skewed nature of the responses, these were collapsed into the never, rarely, or occasionally versus frequently or always range.

The disease self-management efficacy scale was included to gauge individual respondents’ sense of control over the management of their health care [25,26], Respondents were asked about their level of agreement (using a 4-point Likert scale) with the following statements: (1) when all is said and done, I am the person who is responsible for taking care of my health; (2) taking an active role in my own health care is the most important thing that affects my health; (3) I know what each of my prescribed medications do; (4) I am confident that I can tell whether I need to go see the doctor or whether I can take care of a health problem myself; (5) I am confident I can tell a doctor concerns I have even if he or she does not ask; (6) I am confident I can follow through on medical treatments I may need to do at home; (7) I have been able to maintain (keep up with) lifestyle changes such as eating right or exercising; (8) I know how to prevent problems with my health; (9) I am confident I can figure out solutions when new problems arise with my health; and (10) I am confident that I can maintain lifestyle changes like eating right and exercising, even during times of stress. Scores for this scale ranged from 4 to 40, with higher scores indicating higher efficacy.

The use of web-based health information may also reflect barriers to care, reflecting the need for help and support in managing care and treatment. The barriers to self-care scale were measured based on levels of agreement with the following statements: (1) I need help learning what I should be doing to take better care of my health; (2) I need help learning how to take better care of my health in a way that works for me and my life; (3) I do not have the money it takes to do things that will improve my health or condition; (4) I wish I could change and do things that are healthier, but I just do not think I can; and (5) all of my different health problems and conditions make it difficult for me to take better care of myself. Scale values ranged from 5 to 20, with higher scores indicating more barriers.

Patients also seek web-based health information when their experiences with medical providers are frustrating. The health care frustration scale assesses whether participants felt any of the following frustrations [23,24]: (1) felt tired of describing their same conditions and problems every time they went to a hospital or doctor’s office, (2) left the hospital or doctor’s office and felt confused about what they should do, (3) wished their doctor had more time to spend talking with them, (4) felt tired of feeling on their own when it came to taking care of their health problems, (5) felt that their doctor did not realize what it was really like for them at home trying to take care of their health problems, and (6) wished they had a friend or family member who could go to the doctor with them. Responses were coded as “never” (1), “occasionally” (2), or “frequently” (3). Scores for this scale ranged from 6 to 18, with higher scores indicating higher health care frustrations [34].

Finally, respondents might gain knowledge about their chronic condition and insight into their medical condition by participating in a program specifically designed to prevent or treat chronic illnesses [40-43]. For example, the Chronic Disease Self-Management Program (CDSMP) is a universal program that applies to any chronic condition, although disease-specific translations also exist to build skills to manage arthritis, diabetes, chronic pain, and HIV and AIDS [44]. Previous research has indicated that CDSMP improved outcomes while reducing costs [45].

### Technology Use and Credibility

Aside from health concerns and conditions, web-based health information seeking is also a function of the level of comfort in using technology and perceptions regarding the credibility of information found alone. Technology use was measured by whether the respondent had used the following technologies in the past 2 weeks: computer (laptop, desktop, or tablet), smartphone, email (from a computer, smart phone, or tablet), internet (from a computer, smart phone, or tablet), Skype or other video systems (from a computer, smart phone, or tablet), or Facebook or other social media (eg, Twitter). Responses were coded from 0-6 depending on how many of these technologies individual participants reported having used in the past 2 weeks. Perceptions regarding the credibility of web-based information are measured with the question of how reliable they believe information on the internet is about health or medical conditions. Responses were coded from 0 (not at all) to 3 (extremely).

### Data Analysis

All analyses were performed using SPSS version 25 (IBM Corporation). We calculated descriptive statistics for all variables of interest, which were compared across the 2 dependent variables in phase 1. Chi-square tests were used for categorical variables and two-tailed independent sample *t* tests were used for continuous and count variables, after assessing frequency distributions and tests for variance equality. As each dependent variable was dichotomous, we used logistic regression to estimate the models. Model selection was based on stepwise regression using backward elimination of nonsignificant predictor variables. Predictor variables were eliminated when they did not improve the overall model fit, as reflected by the likelihood ratio test. The final regression models included the fewest predictors from the model that provided the best fit to the data. Omnibus tests of model coefficients confirmed no significant loss of variance during backward entry steps for any of the 3 models fitted in this study. However, both full and final reduced regression models are presented in the tables described in the *Results* section. For all analyses, statistical significance was set at *P*<.05.

## Results

### Phase 1 Study Results

Table 1 provides the sample characteristics for the two phase 1 dependent variables. Among the 1922 men who had used the internet in the past week, 57.34% (1102) reported seeking information about a specific disease or medical problem and 50.83% (977) reported seeking information about a medical treatment or procedure. About 58.32% (1121/1922) of the participants were African American and 41.68% (801/1922) were Hispanic. The average age of the sample was 56.63 (SD 10.01) years. The majority of participants attended at least some college (1536/1922, 79.92%), over half were married or partnered (997/1922, 51.87%), and most reported having a routine physician visit in the past year (1627/1922, 84.65%). On average, participants reported living with 2.58 (SD 1.61) other people, having 3.93 (SD 2.9) chronic conditions, and taking 3.39 (SD 2.02) medications daily. About 57.7% (1109/1922) reported that they frequently or always received the help and support needed to improve their health and manage their health problems, and 17.43% (335/1922) reported attending a program to prevent or manage their chronic illness in the past year.

**Table 1:** Sample characteristics by web-based information-seeking behavior (N=1922).

Characteristics			Total (N=1922)		Looked for specific disease or medical problem															Looked for medical treatments and procedures												
					No (n=820)		Yes (n=1102)		Chi-square (*df*)			*t* test (*df*)			*P* value			No (n=945)				Yes (n=977)			Chi-square (*df*)			*t* test *(df)*			*P* value	
Age (years), mean (SD)			56.63 (10.01)		57.77 (10.19)		55.78 (9.79)		N/A^a^			4.33 (1920)			<.001			57.22 (10.13)				56.05 (9.86)			N/A			2.56 (1920)			.01	
**Race or ethnicity, n (%)**										3.1 (1)			N/A			.08										0.3 (1)			N/A			.59
	African American	1121 (58.32)		497 (60.61)		624 (56.62)											557 (58.9)				564 (57.7)											
	Hispanic	801 (41.68)		323 (39.39)		478 (43.38)											388 (41.05)				413 (42.27)											
**Education, n (%)**										20.2 (2)			N/A			<.001										19.3 (2)			N/A			<.001
	High school or less	386 (20.08)		197 (24.02)		189 (17.15)											224 (23.7)				162 (16.58)											
	Some college or 2-year degree	825 (42.92)		359 (43.78)		466 (42.29)											407 (43.06)				418 (42.78)											
	4-year degree or more	711 (36.99)		264 (32.19)		447 (40.56)											314 (33.22)				397 (40.63)											
**Marital status, n (%)**										0.9 (3)			N/A			.82										1.5 (3)			N/A			.68
	Married or partnered	997 (51.87)		427 (52.07)		570 (51.72)											481 (50.89)				516 (52.81)											
	Never married	485 (25.23)		200 (24.39)		285 (25.86)											250 (26.45)				235 (24.05)											
	Divorced or separated	365 (18.99)		162 (19.75)		203 (18.42)											178 (18.83)				187 (19.14)											
	Widowed	75 (3.9)		31 (3.78)		44 (3.99)											36 (3.81)				39 (3.99)											
Persons living in household (including self), mean (SD)			2.58 (1.61)		2.47 (1.59)		2.67 (1.62)		N/A			−2.68 (1920)			.007			2.48 (1.57)				2.68 (1.63)			N/A			−2.76 (1920)			.006	
Number of chronic conditions, mean (SD)			3.93 (2.9)		3.64 (2.76)		4.14 (2.98)		N/A			−3.8 (1920)			<.001			3.6 (2.73)				4.24 (3.02)			N/A			−4.85 (1920)			<.001	
Number of medications taken daily, mean (SD)			3.39 (2.02)		3.36 (2.03)		3.42 (2.01)		N/A			−0.72 (1920)			.47			3.32 (2.05)				3.46 (1.98)			N/A			−1.51 (1920)			.13	
**Routine physician visit in past year, n (%)**										0.2 (1)			N/A			.62										5.2 (1)			N/A			.02
	No	295 (15.35)		122 (14.87)		173 (15.69)											163 (17.24)				132 (13.51)											
	Yes	1627 (84.65)		689 (84.02)		929 (84.3)											782 (82.75)				845 (86.48)											
General health status, mean (SD)			2.85 (0.88)		2.91 (0.87)		2.81 (0.88)		N/A			2.55 (1920)			.01			2.89 (0.87)				2.82 (0.89)			N/A			1.78 (1920)			.08	
**Get the help or support needed to improve health and manage health problems, n (%)**										8.3 (1)			N/A			.004										0.5 (1)			N/A			.48
	Never or rarely or occasionally	813 (42.29)		316 (38.53)		497 (45.09)											392 (41.48)				421 (43.09)											
	Frequently or always	1109 (57.7)		504 (61.46)		605 (54.9)											553 (58.51)				556 (56.91)											
Disease self-management efficacy (Cronbach α=.844), mean (SD)			28.54 (2.58)		28.58 (2.77)		28.5 (2.42)		N/A			0.69 (1920)			.49			28.61 (2.64)				28.47 (2.51)			N/A			1.22 (1920)			.22	
Barriers to self-care (Cronbach α=.844), mean (SD)			11.48 (3.64)		10.96 (3.69)		11.87 (3.55)		N/A			−5.47 (1920)			<.001			11.04 (3.62)				11.91 (3.61)			N/A			−5.26 (1920)			<.001	
Health care frustrations (Cronbach α=.856), mean (SD)			9.45 (3.13)		8.85 (2.93)		9.89 (3.2)		N/A			−7.4 (1920)			<.001			8.86 (2.9)				10.01 (3.24)			N/A			−8.2 (1920)			<.001	
Sources of technology use in past 2 weeks, mean (SD)			4.98 (0.8)		4.87 (0.81)		5.07 (0.78)		N/A			−5.64 (1920)			<.001			4.85 (0.8)				5.12 (0.77)			N/A			−7.46 (1920)			<.001	
Perceived reliability of information received on internet about health or medical conditions, mean (SD)			1.40 (0.69)		1.26 (0.69)		1.51 (0.68)		N/A			−7.75 (1920)			<.001			1.27 (0.68)				1.53 (0.68)			N/A			−8.4 (1920)			<.001	
**Ever attend program to prevent or manage chronic illness in past year, n (%)**										22.4 (1)			N/A			<.001										38.7 (1)			N/A			<.001
	No	1587 (82.57)		716 (87.31)		871 (79.04)											832 (88.04)				755 (77.27)											
	Yes	335 (17.43)		104 (12.68)		231 (20.26)											113 (11.95)				222 (22.72)											

^a^N/A: not applicable.

When comparing sample characteristics by the 2 web-based health information–seeking behaviors (ie, both looked on the internet for information about specific diseases or medical problems and medical treatments and procedures), on average, participants who sought health information on the internet were significantly younger, lived with more people in their household, had more chronic conditions, reported more barriers to self-care, and reported higher health care frustrations. A significantly larger proportion of men who sought web-based health information were more educated and attended a program to prevent or manage their chronic illness in the past year. On average, participants who sought health information on the internet reported using more sources of technology and perceived health and medical information received on the internet to be more reliable.

Table 2 presents the results for seeking web-based information for a specific disease or medical condition among those reporting the use of the internet in the past 2 weeks. Compared with men who did not seek web-based health information for a specific disease or medical condition, men who had some college or a 2-year degree (odds ratio [OR] 1.35, 95% CI 1.04-1.74; *P*=.02), had a 4-year degree or higher (OR 1.91, 95% CI 1.45-2.50; *P*<.001), and attended a program to prevent or manage their chronic illness (OR 1.40, 95% CI 1.07-1.83; *P*=.01) were more likely to seek web-based information for a specific disease or medical condition. For each unit increase in self-reported chronic conditions (OR 1.04, 95% CI 1-1.08; *P*=.03), health care frustrations (OR 1.09, 95% CI 1.05-1.12; *P*<.001), sources of technology used (OR 1.27, 95% CI 1.12-1.44; *P*<.001), and perceived reliability of health and medical information received on the internet (OR 1.70, 95% CI 1.46-1.97; *P*<.001), the odds of seeking information on the internet for a specific disease or medical condition increased. For each unit increase in self-reported health status, the odds of seeking information on the internet for a specific disease or medical condition decreased (OR 0.86, 95% CI 0.76-0.97; *P*=.01).

**Table 2:** Factors associated with looking on the internet for information about a specific disease or medical problem (N=1922)^a^.

Variable			Full model							Reduced model				
			β (SE)		*P* value		OR^b^ (95% CI)		β (SE)			*P* value		OR (95% CI)
Age			−.01 (0.01)		.21		0.99 (0.98-1)		−.01 (0.01)			.09		0.99 (0.98-1)
**Race or ethnicity**														
	African American	—^c^		—		1		—			—		1	
	Hispanic	.16 (0.10)		.12		1.17 (0.96-1.43)		.17 (0.10)			.09		1.19 (0.98-1.45)	
**Education**														
	High school or less	—		—		1		—			—		1	
	Some college or 2-year degree	.29 (0.13)		.03		1.33 (1.03-1.72)		.3 (0.13)			.02		1.35 (1.04-1.74)	
	4-year degree or more	.64 (0.14)		<.001		1.89 (1.43-2.49)		.65 (0.14)			<.001		1.91 (1.45-2.50)	
**Marital status**														
	Married or partnered	—		—		1		N/A^d^			N/A		N/A	
	Never married	−.02 (0.13)		.92		0.99 (0.76-1.28)		N/A			N/A		N/A	
	Divorced or separated	−.02 (0.14)		.89		0.98 (0.75-1.28)		N/A			N/A		N/A	
	Widowed	.20 (0.26)		.44		1.22 (0.74-2.03)		N/A			N/A		N/A	
Persons living in household (including self)			.04 (0.03)		.25		1.04 (0.97-1.11)		N/A			N/A		N/A
Number of chronic conditions			.04 (0.02)		.049		1.04 (1-1.08)		.04 (0.02)			.03		1.04 (1-1.08)
Number of medications taken daily			.01 (0.03)		.63		1.01 (0.96-1.07)		N/A			N/A		N/A
**Routine physician visit in past year**														
	No	—		—		1		N/A			N/A		N/A	
	Yes	.06 (0.15)		.69		1.06 (0.8-1.42)		N/A			N/A		N/A	
General health status			−.13 (0.07)		.046		0.88 (0.77-1)		−.15 (0.06)			.01		0.86 (0.76-0.97)
**Get the help or support needed**														
	Never or rarely or occasionally	—		—		1		N/A			N/A		N/A	
	Frequently or always	−.14 (0.11)		.22		0.87 (0.7-1.09)		N/A			N/A		N/A	
Disease self-management efficacy			.01 (0.02)		.64		1.01 (0.97-1.05)		N/A			N/A		N/A
Barriers to self-care			.01 (0.02)		.38		1.01 (0.98-1.05)		N/A			N/A		N/A
Health care frustrations			.07 (0.02)		<.001		1.07 (1.03-1.12)		.08 (0.02)			<.001		1.09 (1.05-1.12)
Sources of technology use in past 2 weeks			.24 (0.06)		<.001		1.27 (1.12-1.44)		.24 (0.06)			<.001		1.27 (1.12-1.44)
Perceived reliability of information received on internet about health or medical conditions			.54 (0.08)		<.001		1.72 (1.48-1.99)		.53 (0.08)			<.001		1.7 (1.46-1.97)
**Ever attend program to prevent or manage chronic illness in past year**														
	No	—		—		1		—			—		1	
	Yes	.31 (0.14)		.02		1.37 (1.04-1.79)		.34 (0.14)			.01		1.4 (1.07-1.83)	

^a^Nagelkerke *R*^2^=0.122 for full model; Nagelkerke *R*^2^=0.119 (8 iterations) for reduced model.

^b^OR: odds ratio.

^c^Not available; referent category for independent variables.

^d^N/A: not applicable; referent category for dependent variable (not looking on the internet for information about a specific disease or medical problem).

Table 3 presents the results for seeking web-based information for medical treatments and procedures among those reporting having used the internet in the past 2 weeks. Compared with men who did not seek web-based information about medical treatments and procedures, men who had a college education or a 2-year degree (OR 1.32, 95% CI 1.02-1.72; *P*=.03), had a 4-year degree or higher (OR 1.72, 95% CI 1.31-2.25; *P*<.001), attended a routine physician visit in the past year (OR 1.48, 95% CI 1.13-1.94; *P*=.004), and attended a program to prevent or manage their chronic illness (OR 1.59, 95% CI 1.22-2.08; *P*=.001) were more likely to seek web-based information about medical treatments and procedures. For each unit increase in self-reported chronic conditions (OR 1.06, 95% CI 1.02-1.1; *P*=.001), health care frustrations (OR 1.12, 95% CI 1.09-1.16; *P*=.001), sources of technology used (OR 1.44, 95% CI 1.27-1.63; *P*<.001), and the perceived reliability of health and medical information received on the internet (OR 1.69, 95% CI 1.46-1.95; *P*<.001), the odds of seeking web-based information about medical treatments and procedures increased.

**Table 3:** Factors associated with seeking on the internet information about medical treatments and procedures (N=1922)^a,b^.

Variable			Full model							Reduced model				
			β (SE)		*P* value		OR^c^ (95% CI)		β (SE)			*P* value		OR (95% CI)
Age			0 (0.01)		.77		1 (0.99-1.01)		N/A^d^			N/A		N/A
**Race or ethnicity**														
	African American	—^e^		—		1		N/A			N/A		N/A	
	Hispanic	.05 (0.1)		.61		1.05 (0.86-1.29)		N/A			N/A		N/A	
**Education**														
	High school or less	—		—		1		—			—		1	
	Some college or 2-year degree	.28 (0.13)		.04		1.33 (1.02-1.72)		.28 (0.13)			.03		1.32 (1.02-1.72)	
	4-year degree or more	.54 (0.14)		<.001		1.72 (1.3-2.26)		.54 (0.14)			<.001		1.72 (1.31-2.25)	
**Marital status**														
	Married or partnered	—		—		1		N/A			N/A		N/A	
	Never married	−.21 (0.13)		.13		0.82 (0.63-1.06)		N/A			N/A		N/A	
	Divorced or separated	.05 (0.14)		.70		1.05 (0.81-1.38)		N/A			N/A		N/A	
	Widowed	.04 (0.26)		.89		1.04 (0.63-1.72)		N/A			N/A		N/A	
Persons living in household (including self)			.04 (0.03)		.24		1.04 (0.97-1.11)		N/A			N/A		N/A
Number of chronic conditions			.05 (0.02)		.006		1.05 (1.02-1.1)		.06 (0.02)			.001		1.06 (1.02-1.1)
Number of medications taken daily			0 (0.03)		.99		1 (0.95-1.06)		N/A			N/A		N/A
**Routine physician visit in past year**														
	No	—		—		1		—			—		1	
	Yes	.40 (0.15)		.007		1.49 (1.11-1.99)		.39 (0.14)			.004		1.48 (1.13-1.94)	
General health status			−.07 (0.07)		.32		0.94 (0.83-1.07)		N/A			N/A		N/A
**Get the help or support needed**														
	Never or rarely or occasionally	—		—		1		N/A			N/A		N/A	
	Get the help or support needed: frequently or always	.04 (0.11)		.70		1.04 (0.84-1.3)		N/A			N/A		N/A	
Disease self-management efficacy			−.01 (0.02)		.52		0.99 (0.95-1.03)		N/A			N/A		N/A
Barriers to self-care			.02 (0.02)		.32		1.02 (0.98-1.05)		N/A			N/A		N/A
Health care frustrations			.1 (0.02)		<.001		1.11 (1.07-1.15)		.12 (0.02)			<.001		1.12 (1.09-1.16)
Sources of technology use in past 2 weeks			.37 (0.06)		<.001		1.45 (1.28-1.65)		.36 (0.06)			<.001		1.44 (1.27-1.63)
Perceived reliability of information received on internet about health or medical conditions			.56 (0.08)		<.001		1.75 (1.51-2.03)		.53 (0.07)			<.001		1.69 (1.46-1.95)
**Ever attend program to prevent or manage chronic illness in past year**														
	No	—		—		1		—			—		1	
	Yes	.44 (0.14)		.001		1.56 (1.19-2.04)		.47 (0.14)			.001		1.59 (1.22-2.08)	

^a^Nagelkerke *R*^2^=0.155 for full model; Nagelkerke *R*^2^=0.148 (10 iterations) for reduced model.

^b^The same dependent variable and referent category is used for the full and reduced models.

^c^OR: odds ratio.

^d^N/A: not applicable.

^e^Not available; referent category for independent variables.

### Phase 2 Study Results

Table 4 presents phase 2 results for the 1035 participants who discussed what they found on the internet with their medical providers among those who had a routine physician visit in the past year. Relative to the 71.4% (739/1035) of men who reported both web-based health information seeking behaviors (ie, disease-specific information and medical treatments or procedures), men who only looked for information about specific diseases on the internet were significantly less likely to discuss what they found with their medical provider (OR 0.52, 95% CI 0.37-0.74; *P*<.001). Relative to men who did not discuss their web-based findings with medical providers, men who were Hispanic (OR 1.41, 95% CI 1.09-1.83; *P*<.001) and attended a program to prevent or manage their chronic illness (OR 2.19, 95% CI 1.61-2.98; *P*<.001) were more likely to discuss the web-based findings with their medical provider. For each unit increase in the number of medications taken daily (OR 1.13, 95% CI 1.05-1.21; *P*=.001), barriers to self-care (OR 1.04, 95% CI 1-1.08; *P*=.04), and sources of technology used (OR 1.24, 95% CI 1.05-1.46; *P*=.01), the odds of discussing web-based information with medical providers increased.

**Table 4:** Factors associated with discussing online information with medical providers (n=1035)^a,b^.

Variable		Full model				Reduced model		
		β (SE)	*P* value	OR^c^ (95% CI)	β (SE)		*P* value	OR (95% CI)
**Looked on the internet for health information**								
	Both	—^d^	—	1	—		—	1
	Only about medical treatments and procedures	−.35 (0.22)	.11	0.7 (0.45-1.09)	−.37 (0.22)		.09	0.69 (0.45-1.06)
	Only about specific diseases of medical problems	−.6 (0.18)	.001	0.55 (0.39-0.79)	−.65 (0.18)		<.001	0.52 (0.37-0.74)
Age		0 (0.01)	.77	1 (0.99-1.02)	N/A^e^		N/A	N/A
**Race or ethnicity**								
	African American	—	—	1	—		—	1
	Hispanic	.36 (0.14)	.01	1.43 (1.09-1.87)	.35 (0.13)		.01	1.41 (1.09-1.83)
**Education**								
	High school or less	—	—	1	N/A		N/A	N/A
	Some college or 2-year degree	.23 (0.19)	.24	1.25 (0.86-1.83)	N/A		N/A	N/A
	4-year degree or more	.17 (0.2)	.39	1.19 (0.8-1.76)	N/A		N/A	N/A
**Marital status**								
	Married or partnered	—	—	1	N/A		N/A	N/A
	Never married	−.11 (0.18)	.56	0.9 (0.63-1.28)	N/A		N/A	N/A
	Divorced or separated	−.13 (0.19)	.51	0.88 (0.61-1.28)	N/A		N/A	N/A
	Widowed	−.06 (0.34)	.86	0.94 (0.49-1.82)	N/A		N/A	N/A
Persons living in household (including self)		0 (0.05)	.99	1 (0.91-1.1)	N/A		N/A	N/A
Number of chronic conditions		.01 (0.02)	.73	1.01 (0.96-1.06)	N/A		N/A	N/A
Number of medications taken daily		.11 90.04)	.003	1.12 (1.04-1.21)	.12 (0.04)		.001	1.13 (1.05-1.21)
General health status		.1 (0.09)	.26	1.1 (0.93-1.31)	.15 (0.08)		.07	1.16 (0.99-1.35)
**Get the help or support needed**								
	Never or rarely or occasionally	—	—	1	N/A		N/A	N/A
	Frequently or always	.21 (0.15)	.16	1.23 (0.92-1.65)	N/A		N/A	N/A
Disease self-management efficacy		.02 (0.03)	.43	1.02 (0.97-1.08)	N/A		N/A	N/A
Barriers to self-care		.04 (0.02)	.1	1.04 (0.99-1.09)	.04 (0.02)		.04	1.04 (1-1.08)
Health care frustrations		.04 (0.03)	.11	1.04 (0.99-1.09)	N/A		N/A	N/A
Sources of technology use in past 2 weeks		.2 (0.09)	.02	1.22 (1.03-1.45)	.22 (0.09)		.01	1.24 (1.05-1.46)
Perceived reliability of information received on internet about health or medical conditions		.15 (0.1)	.15	1.16 (0.95-1.41)	N/A		N/A	N/A
**Ever attend program to prevent or manage chronic illness in past year**								
	No	—	—	1	—		—	1
	Yes	.7 (0.16)	<.001	2.01 (1.46-2.77)	.78 (0.16)		<.001	2.19 (1.61-2.98)

^a^Nagelkerke *R*^2^=0.112 for full model; Nagelkerke *R*^2^=0.101 (10 iterations) for reduced model.

^b^The same dependent variable and referent category is used for the full and reduced models.

^c^OR: odds ratio.

^d^Not available; referent category for independent variables.

^e^N/A: not applicable.

## Discussion

### Principal Findings

This study examined health information seeking among a uniquely vulnerable and intersectional population, African American and Hispanic men aged 40 years and older with one or more chronic conditions. The specific results are worth discussing. First, internet-based health information is an important tool for African American and Hispanic men to use to learn about a specific disease or medical problem as well as medical treatments and procedures and to foster patient-provider conversations about these health-related internet searches, as illustrated by about half of the sample looking for information on the internet. Similar to previous studies, our study suggests that men who are younger [46-48], more highly educated [47,48], use technology more often [49], and believe the internet to be a reliable source [50] report seeking web-based health information in the past year to learn about a specific disease or medical treatment.

Those with more chronic conditions and greater health care frustrations were more likely to use the internet for both purposes (ie, to learn about a specific disease and medical treatments). Previous studies have demonstrated that people living with chronic conditions rely on the internet for help and support and might seek to learn about other people’s experiences about a disease through web-based discussions [39]. People living with chronic conditions who experienced health care–related frustrations from unfulfilled needs in a medical encounter have been known to report greater functional limitations and greater self-care barriers to manage their condition or disease [51]. Internet-based health information could be used to help meet those needs.

Men who attended a disease prevention or management program in the past year were more likely to look on the internet to learn about a specific disease or medical problem and to learn about medical treatments and procedures. Considering that these evidence-based programs help increase participants’ health behaviors and self-efficacy [52], program participants may feel encouraged to seek additional information to better understand the content of their program and their disease. Both web-based and traditional CDSMPs may also provide links, videos, and other resources to supplement the course materials, thereby encouraging program participants to seek this information on the internet as part of their disease self-management.

Interestingly, compared with men who used the internet only to learn about a specific disease or condition, men who reported both web-based health information–seeking behaviors were more likely to speak to their medical professional about what they found on the internet. It is possible that those who search on the web only to learn about a specific disease no longer feel the need to consult a physician, consequently substituting routine care [53]. Men who seek information on both diseases and treatments may have greater concerns about the credibility of web-based information or about their ability to evaluate this information [53]. Those who searched for treatments and procedures in addition to the general condition may also be exposed to web-based medical advertisements that encourage them to speak to their doctor about these treatment options [53-56]. Through their internet searches, patients reported increased confidence, control, and comfort in discussing their condition and treatments with their medical provider [57]; enhanced understanding of the medical jargon [58]; and satisfaction of feeling better informed [54]. Hispanic men more frequently discussed what they found on the internet with medical professionals. Studies suggest that web-based health information seeking gives Hispanic patients the confidence to discuss their health concerns with their doctors [30]. In the recent study by Camacho-Rivera et al [59] with a large representative sample of Hispanic adults in the United States, the authors found that Hispanics trusted cancer information from their doctors a lot (1014/1512, 67.06%) compared with information from the internet (309/1512, 20.44%). Although there was an important increase in trusting cancer information on the internet from 2014 to 2018, doctors remained the most trusted source of health information for Hispanics [59]. This study supports our findings that Hispanic men were more likely to talk with medical professionals about their web-based health searches.

Men with chronic conditions who had better general health statuses reported communicating with their medical professionals about what they found on the internet. This result contradicts previous studies [55], which suggest that those in poor health are more likely to talk to their medical providers about their web-based health information seeking than those in good health. However, higher medication intake is associated with poorer health (eg, frailty, disability, and fall risk) [60,61]. Poor health status can also lead to greater self-care barriers [62]. Those who take more medications daily also report more barriers to managing their chronic conditions [50]. Medication and self-care barriers were highlighted in our study as factors associated with discussing web-based health information with a medical professional.

It is possible that those with better health status, those who take more medications daily, and those with more self-care barriers may seek medical care more than once a year. It is known that increased physician visits to stay healthy and to get help to manage chronic conditions [63] may provide greater opportunities to discuss web-based health information–seeking behaviors. Increased visits may lead to better patient-physician interactions where bringing internet-based health information would not be seen as a threat but rather as something to be encouraged [64-66].

### Limitations

This study has several limitations. The cross-sectional nature of this study did not allow for the assessment of causal relationships over time. On the basis of the funding mechanism supporting this study, data were only collected from African American and Hispanic men aged 40 years and older with one or more chronic conditions. Although these subgroups often report health-related disparities, additional insights might have been gained, including men of other races and ethnicities (eg, non-Hispanic White, Asian or Pacific Islander, and American Indian or Alaska Native). We hope that future research will expand the scope of this study and provide additional comparisons. This study excludes African American and Hispanic males with one or more chronic conditions who do not have access to the internet. This digital divide continues to disproportionately impact the health of minorities and contribute to social inequalities in the United States [67]. In addition, no information was gathered about health literacy, the types of web-based information sources they used, or the credibility of these information sources (eg, government websites). Future research on internet-based health information–seeking behaviors of African American and Hispanic men with chronic conditions should consider assessing the health literacy level of respondents as well as their knowledge of credible health information sources [68]. In addition, in this study, African American and Hispanic subgroups were included in the analyses. Given the potential differences across racial or ethnic subgroups in terms of sociodemographics, behaviors, perceptions, and health care use, future studies may consider performing analyses on these subgroups separately or making direct comparisons between them.

### Conclusions

Overall, this study provides an overview of health information–seeking behaviors among African American and Hispanic men with chronic conditions. Understanding these factors is crucial to influencing internet-based health communication, improving patient-provider communication, and ultimately improving the care and treatment of African American and Hispanic men.

## Abbreviations

CDSMP: Chronic Disease Self-Management Program
OR: odds ratio
